# Mitral and Tricuspid Annular Abnormalities in Hypereosinophilic Syndrome—Insights from the Three-Dimensional Speckle-Tracking Echocardiographic MAGYAR-Path Study

**DOI:** 10.31083/j.rcm2404115

**Published:** 2023-04-17

**Authors:** Attila Nemes, Árpád Kormányos, Gergely Rácz, Nóra Ambrus, Imelda Marton, Zita Borbényi

**Affiliations:** ^1^Department of Medicine, Albert Szent-Györgyi Medical School, University of Szeged, 6725 Szeged, Hungary; ^2^Division of Haematology, Department of Medicine, Albert Szent-Györgyi Medical School, University of Szeged, 6725 Szeged, Hungary; ^3^Department of Transfusiology, Albert Szent-Györgyi Medical School, University of Szeged, 6725 Szeged, Hungary

**Keywords:** annulus, echocardiography, hypereosinophilic syndrome, mitral, speckle-tracking, three-dimensional, tricuspid

## Abstract

**Background:**

Hypereosinophilic syndrome (HES) is a peripheral eosinophilia characterized by elevated absolute eosinophil cell count (>1.500 cells/μL) and consequent tissue and end-organ damage. Our aim was to evaluate the mitral annular (MA) and/or tricuspid annular (TA) parameters of patients with HES and to determine whether there are any changes in these parameters compared to healthy individuals.

**Methods:**

17 patients with HES were involved in our study, 2 cases were excluded due to suboptimal image quality (mean age of the evaluated patients: 61.7 ± 11.2 years, 10 males). Their data were compared with those of 24 healthy subjects (mean age: 55.2 ± 7.9 years, 12 males) in the control group. Complete echocardiographic examinations were performed including two-dimensional (2D) Doppler echocardiography and three-dimensional echocardiography (3DE) to assess the MA and the TA.

**Results:**

Comparing the echocardiographic parameters of the HES patients with those of the healthy volunteers, the following changes were seen: the interventricular septum was significantly thickened in HES patients, no other significant changes were detected between the examined patient groups. End-diastolic and end-systolic MA diameters, areas and perimeters were increased and MA fractional area change and MA fractional shortening were decreased in HES patients. From TA morphological parameters, only end-diastolic TA area and end-systolic TA perimeter were significantly increased in HES patients. Functional TA parameters showed no significant alterations in the HES group. In patients with HES, no correlations could be detected between 2D and 3D echocardiographic data with the examined laboratory findings.

**Conclusions:**

The extent of the dilation of the MA is more pronounced than that of the TA in HES. MA functional impairment is present in HES.

## 1. Introduction

Hypereosinophilic syndrome (HES) is a peripheral eosinophilia featured by elevated absolute eosinophil cell count (>1.500 cells/μL) and consequent tissue and end-organ damage, including the heart, lungs, nervous system and gastrointestinal tract [1, 2]. Cardiac impairment was seen in some cases, early phases were characterized by eosinophilic infiltration, then there was a thrombotic stage, followed by a final fibrotic stage [3, 4, 5, 6]. Novel cardiovascular imaging techniques enabled the detection of subclinical cardiac abnormalities in HES cases being in necrotic phase including dilation and functional impairment of both atria [7, 8] and reduction of left ventricular (LV) rotational and deformation mechanics [9, 10]. Based on our results, it can be hypothesized that the abnormalities of the ventricular and atrial volumetric and deformation parameters lead to compensatory changes in the mitral (MA) and/or tricuspid annulus (TA), in their size and function. Therefore, characteristics of MA/TA were assessed by the novel non-invasive three-dimensional (3D) echocardiography.

## 2. Materials and Methods

### 2.1 Patient Population

There were 17 subjects in the HES group and 24 patients in the group of healthy controls. HES patients were recruited between May 2012 and December 2021 prospectively in the Division of Haematology at the University of Szeged. Two patients were excluded from study in the HES group due to suboptimal image quality with 3D echocardiography. From the 15 patients participating in the study, 14 patients had idiopathic HES and 1 patient had HES associated with acute T-lymphoma (mean age: 61.7 ± 11.2 years, 10 males). The 24 healthy subjects were volunteers (mean age: 55.2 ± 7.9 years, 12 males), who had no symptoms, did not have any known cardiovascular risk factors or disorders or were taking any medications. None of the HES patients had any hypereosinophilia-related symptoms at baseline. Only some subjects had previous cardiovascular events including non-ST-elevation myocardial infarction (NSTEMI) (n = 1), deep vein thrombosis in the lower extremity (n = 1) and bilateral iliofemoral embolism (n = 1). All patients in the HES and in the control groups were in sinus rhythm without pulmonary embolism, chronic obstructive pulmonary disease or tumors in the past medical history. Regarding the classic cardiovascular risk factors in the HES patients, 1 patient was treated with diabetes mellitus, 8 patients had hypertension and 4 patients were diagnosed with hypercholesterolaemia. HES patients showed several extracardiac manifestations as well including eosinophilic asthma bronchiale (n = 1), eosinophilic dermatitis (n = 1), duodenal eosinophilia (n = 1), granulomatous necrotizing vasculitis and sensory-motor neuropathy with pulmonary involvement (n = 1), tissue (pulmonary) eosinophilia (n = 1), sole skin involvement (n = 1), and acute T-lymphoma associated hypereosinophilia with gastrointestinal involvement (n = 1). Two-dimensional (2D) and 3D echocardiography extended with Doppler assessments were completed in all subjects. The present substudy served as a part of Motion Analysis of the heart and Great vessels by three-dimensional speckle-tracking echocardiography in Pathological cases (MAGYAR-Path) Study evaluating echocardiographic parameters, including valvular annular parameters using 3D (speckle-tracking) echocardiography in various disorders. Ethical approval was provided by the Institutional and Regional Human Biomedical Research Committee of University of Szeged, Hungary (No.: 71/2011 and updated versions) and our study conducted in accordance with the ethical guidelines of the Declaration of Helsinki (1975 and updated versions). All HES patients and healthy controls gave an informed consent.

### 2.2 2D Doppler Echocardiography

2D gray-scale images were performed using a broadband PST-30BT phased-array transducer (1–5 MHz) with a Toshiba Artida® (Toshiba Medical Systems, Tokyo, Japan) echocardiographic tool. The following echocardiographic parameters were measured in all patients: diameter of the left atrium, diameter and volume of the LV respecting the cardiac cycle, thickness of the interventricular septum and thickness of the LV posterior wall, LV ejection fraction measured using the Simpson’s method [11]. With pulsed Doppler transmitral flow velocities were measured in diastole and their ratio (E/A) were calculated. With tissue Doppler imaging E/E’ was measured as a ratio of early diastolic transmitral flow velocity (E) and mitral annular velocity (E’). With Doppler echocardiography tricuspid regurgitation pressure gradient was estimated, as well.

### 2.3 Three-Dimensional (Speckle-Tracking) Echocardiography

3D data acquisitions were completed by a Toshiba ${\mathrm{Artida}}^{\text{TM}}$ (Toshiba Medical Systems, Tokyo, Japan) cardiac ultrasound tool with a PST-25SX matrix-array transducer. From apical window, 6 subvolumes were collected in 6 heart cycles within a single breath-hold, then 3D Wall Motion Tracking software version 2.7 (Toshiba Medical Systems, Tokyo, Japan) was used for offline image analysis on the automatically created full volume 3D dataset [12, 13, 14]. Left atrium (LA) and LV dimensions were assessed using the parasternal long-axis view. LA volumes were evaluated from apical 2-chamber and 4-chamber longitudinal views, and the measured values were indexed to body surface area.

### 2.4 3D Echocardiography-Derived MA/TA Measurements

**Fig. 1.**
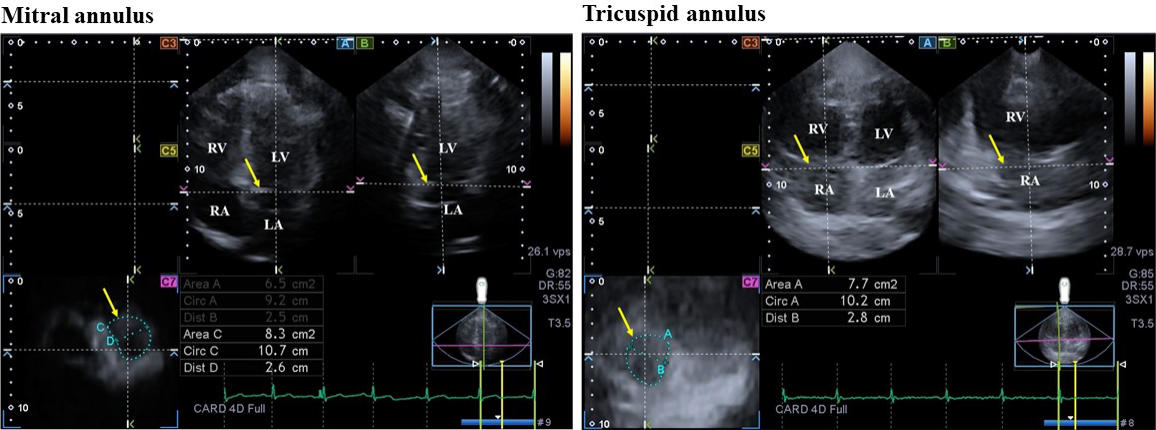
**Three-dimensional (3D) echocardiographic assessment of mitral and tricuspid annuli in a patient with hypereosinophilic syndrome**. (A) apical four-chamber view, (B) apical two-chamber view and cross-sectional view (C7) of the mitral and tricuspid annuli optimalised on mitral and tricuspid annular images. Yellow arrows indicate plane of the mitral and tricuspid annuli. Abbreviations: Area, mitral/tricuspid annular area; Circ, mitral/tricuspid annular perimeter; Dist, mitral/tricuspid annular diameter; LA, left atrium; LV, left ventricle; RA, right atrium; RV, right ventricle.

The end-diastole was considered when peak R wave was on electrocardiogram. The end-systole was considered as the first frame when the aortic valve was closed (at the end of T wave on electrocardiogram). MA/TA assessments were made using optimized image planes on the endpoints of the MA/TA on apical two- and four-chamber views and on C7 short-axis view. Several MA/TA measures and features of their function were calculated at end-diastole and at end-systole (Fig. 1) [15, 16]:

MA/TA dimensions:

∙ MA/TA diameter: perpendicular line connecting the peak of MA/TA curvature and the middle of the straight MA/TA border,

∙ MA/TA area: measured by planimetry,

∙ MA/TA perimeter: measured by planimetry.

MA/TA functional properties:

∙ MA/TA fractional shortening (MAFS/TAFS) = [end-diastolic MA/TA diameter – end-systolic MA/TA diameter]/end-diastolic MA/TA diameter × 100,

∙ MA/TA fractional area change (MAFAC/TAFAC) = [end-diastolic MA/TA area – end-systolic MA/TA area]/end-diastolic MA/TA area × 100.

### 2.5 Statistical Analysis

While categorical data were expressed in counts and percentages (%), continuous variables were demonstrated in mean ± standard deviation format. Levene’s test was used for testing homogeneity of variances. Student’s *t*-test was used in case of normally distributed data, while non-normally distributed data were analyzed with Mann-Whitney Wilcoxon test. Chi-squared test and Fisher’s exact test were performed for statistical analysis of categorical variables. Pearson’s correlation coefficients were used to evaluate correlations. For intraobserver and interobserver correlations, intraclass correlation coefficients (ICCs) were calculated [17]. The Bland–Altman method was used to determine intraobserver and interobserver agreements [18]. Multivariable regression analysis with covariable age, hypertension, hyperlipidemia and presence of HES was used for assessment of independent predictors of reduced MAFAC and MAFS. All statistical tests were two-sided. Statistical significance was considered in case of *p *< 0.05. Statistical analyses were performed using the MedCalc software package (MedCalc, Inc., Mariakerke, Belgium).

## 3. Results

### 3.1 Clinical Data

The incidence of hypertension and hyperlipidaemia was higher in the HES group, other demographic parameters were similar between the groups examined (Table 1).

**Table 1.** **Baseline demographic and two-dimensional echocardiographic data of patients with hypereosinophilic syndrome and healthy controls**.

	Controls (n = 24)	HES patients (n = 15)	*p* value
Clinical data			
Age (years)	55.2 ± 7.9	61.7 ± 11.2	0.08
Male gender (%)	12 (50)	10 (67)	0.34
Hypertension (%)	0 (0)	8 (53)	0.0001
Diabetes mellitus (%)	0 (0)	1 (7)	0.38
Hyperlipidaemia (%)	0 (0)	4 (27)	0.02
2D echocardiography			
LA diameter (mm)	38.4 ± 3.2	42.1 ± 7.4	0.15
LA volume index (mL/$m^{2}$)	27.1 ± 7.7	31.3 ± 14.5	0.17
LV end-diastolic diameter (mm)	48.8 ± 3.2	51.7 ± 11.6	0.27
LV end-diastolic volume (mL)	110.7 ± 20.8	116.0 ± 48.9	0.65
LV end-systolic diameter (mm)	31.8 ± 2.9	34.5 ± 11.9	0.31
LV end-systolic volume (mL)	38.6 ± 8.1	42.9 ± 22.1	0.41
Interventricular septum (mm)	9.5 ± 1.2	10.8 ±1.3	0.008
LV posterior wall (mm)	9.8 ± 1.6	9.7 ± 1.3	0.93
LV ejection fraction (%)	65.0 ± 4.4	63.4 ± 9.4	0.48
E (cm/s)	69.3 ± 18.5	76.8 ± 20.1	0.39
A (cm/s)	71.7 ± 21.7	72.7 ± 20.1	0.92
E/A	1.0 ± 0.3	1.1 ± 0.4	0.50
E/E’	9.4 ± 1.2	10.2 ± 1.0	0.46
Tricuspid regurgitation pressure gradient (mm Hg)	15.8 ± 2.2	15.4 ± 2.5	0.93

Abbreviations: 2D, two-dimensional; HES, hypereosinophilic syndrome; E and A, early and late diastolic transmitral flow velocities; E’, early diastolic mitral annular velocity; LA, left atrium; LV, left ventricular.

### 3.2 Laboratory Findings

Absolute eosinophil count (7.4 ± 4.4 × ${\mathrm{10}}^{9}$/L vs. 0.2 ± 0.2 × ${\mathrm{10}}^{9}$/L, *p* = 0.03), eosinophil ratio (45.2 ± 16.5% vs. 3.1 ± 2.1%, *p* = 0.01) and white blood cell count (16.2 ± 6.1 × ${\mathrm{10}}^{9}$/L vs. 6.3 ± 0.9 × ${\mathrm{10}}^{9}$/L, *p* = 0.02) were significantly increased in HES patients compared to that of controls. Other laboratory findings including red blood cell count (4.1 ± 0.6 T/L vs. 4.2 ± 0.3 T/L, *p* = 0.81), hemoglobin (125.0 ± 16.9 g/L vs. 129.1 ± 9.1 g/L, *p* = 0.80), platelet count (272.3 ± 156.1 × ${\mathrm{10}}^{9}$/L vs. 271.9 ± 155.2 × ${\mathrm{10}}^{9}$/L, *p* = 0.85) and hematocrit (36.5 ± 4.1% vs. 36.9 ± 5.3%, *p* = 0.88) did not differ between the groups.

### 3.3 2D Doppler Echocardiographic Data

Table 1 was used for demonstration of 2D echocardiographic parameters of HES patients and controls. Only interventricular septum was significantly thickened in patients with HES, no other parameter differed significantly between the groups examined. Only one HES patient showed grade 2 mitral regurgitation, other HES patients and controls did not show larger than grade 1 valvular insufficiency or had significant valvular stenoses.

### 3.4 3D Echocardiographic MA and TA Data

Increased end-diastolic and end-systolic MA diameters, areas and perimeters together with reduced MAFAC and MAFS could be detected in HES patients as compared to those of controls. From TA morphological parameters, only end-diastolic TA area and end-systolic TA diameter were significantly increased in HES patients who had preserved TA functional parameters (Tables 2,3). Comparative analysis of MA and TA parameters was done between HES cases with vs. without hypertension without significant differences between these parameters (Table 4).

**Table 2.** **Mitral annular data as assessed by three-dimensional echocardiography between hypereosinophilic patients and controls**.

		Controls (n = 24)	HES patients (n = 15)	*p* value
Morphological parameters				
	end-diastolic MA diameter (cm)	2.4 ± 0.3	2.6 ± 0.3	0.03
	end-diastolic MA area (${\mathrm{cm}}^{2}$)	7.5 ± 1.9	9.5 ± 2.3	0.01
	end-diastolic MA perimeter (cm)	10.4 ± 1.3	11.6 ± 1.7	0.03
	end-systolic MA diameter (cm)	1.7 ± 0.3	2.2 ± 0.2	0.001
	end-systolic MA area (${\mathrm{cm}}^{2}$)	3.8 ± 1.1	6.7 ± 2.0	0.001
	end-systolic MA perimeter (cm)	7.4 ± 1.1	9.9 ± 1.9	0.001
Functional parameters				
	MAFAC (%)	47.7 ± 15.8	29.6 ± 13.0	0.001
	MAFS (%)	28.9 ± 12.7	16.6 ± 11.9	0.004

Abbreviations: HES, hypereosinophilic syndrome; MA, mitral annulus; MAFAC, mitral annular fractional area change; MAFS, mitral annular fractional shortening.

**Table 3.** **Tricuspid annular data evaluated by three-dimensional echocardiography between hypereosinophilic patients and controls**.

		Controls (n = 24)	HES patients (n = 15)	*p* value
Morphological parameters				
	end-diastolic TA diameter (cm)	2.3 ± 0.3	2.6 ± 0.5	0.08
	end-diastolic TA area (${\mathrm{cm}}^{2}$)	7.5 ± 1.9	9.1 ± 2.4	0.04
	end-diastolic TA perimeter (cm)	10.7 ± 1.3	11.1 ± 2.0	0.5
	end-systolic TA diameter (cm)	1.9 ± 0.3	2.3 ± 0.4	0.01
	end-systolic TA area (${\mathrm{cm}}^{2}$)	5.9 ± 1.8	6.9 ± 2.1	0.1
	end-systolic TA perimeter (cm)	9.3 ± 1.4	9.9 ± 1.2	0.2
Functional parameters				
	TAFAC (%)	22.6 ± 11.6	23.4 ± 13.6	0.9
	TAFS (%)	17.9 ± 7.4	13.2 ± 7.5	0.08

Abbreviations: HES, hypereosinophilic syndrome; TA, tricuspid annulus; TAFAC, tricuspid annular fractional area change; TAFS, tricuspid annular fractional shortening.

**Table 4.** **Comparison of mitral and tricuspid annular data between hypereosinophilic patients with vs. without hypertension and controls**.

		Controls (n = 24)	HES patients with hypertension (n = 8)	HES patients without hypertension (n = 7)
Morphological MA parameters				
	end-diastolic MA diameter (cm)	2.4 ± 0.3	2.6 ± 0.3	2.7 ± 0.3*
	end-diastolic MA area (${\mathrm{cm}}^{2}$)	7.5 ± 1.9	9.4 ± 2.7*	9.6 ± 2.0*****
	end-diastolic MA perimeter (cm)	10.4 ± 1.3	11.7 ± 1.8**	11.4 ± 1.5
	end-systolic MA diameter (cm)	1.7 ± 0.3	2.2 ± 0.2***	2.1 ± 0.3***
	end-systolic MA area (${\mathrm{cm}}^{2}$)	3.8 ± 1.1	6.5 ± 2.0***	6.8 ± 2.2***
	end-systolic MA perimeter (cm)	7.4 ± 1.1	10.1 ± 1.9***	9.6 ± 2.1***
Functional MA parameters				
	MAFAC (%)	47.7 ± 15.8	29.9 ± 14.0****	29.2 ± 16.0****
	MAFS (%)	28.9 ± 12.7	14.8 ± 10.8***	18.5 ± 15.1
Morphological TA parameters				
	end-diastolic TA diameter (cm)	2.3 ± 0.3	2.5 ± 0.4	2.8 ± 0.6*****
	end-diastolic TA area (${\mathrm{cm}}^{2}$)	7.5 ± 1.9	8.3 ± 1.9	10.0 ± 2.7****
	end-diastolic TA perimeter (cm)	10.7 ± 1.3	10.3 ± 2.2	12.1 ± 1.3*****
	end-systolic TA diameter (cm)	1.9 ± 0.3	2.2 ± 0.4	2.4 ± 0.4*
	end-systolic TA area (${\mathrm{cm}}^{2}$)	5.9 ± 1.8	6.4 ± 1.8	7.5 ± 2.5
	end-systolic TA perimeter (cm)	9.3 ± 1.4	9.5 ± 0.9	10.2 ± 1.5
Functional TA parameters				
	TAFAC (%)	22.6 ± 11.6	22.0 ± 15.4	25.0 ± 12.2
	TAFS (%)	17.9 ± 7.4	12.6 ± 5.7	13.8 ± 9.5

Abbreviations: HES, hypereosinophilic syndrome; MA, mitral annulus; MAFAC, mitral annular fractional area change; MAFS, mitral annular fractional shortening; TA, tricuspid annulus; TAFAC, tricuspid annular fractional area change; TAFS, tricuspid annular fractional shortening. *p = 0.04 vs. Controls; **p = 0.03 vs. Controls; ***p = 0.001 vs. Controls; ****p = 0.01 vs. Controls; *****p = 0.02 vs. Controls.

### 3.5 Correlations and Regression Analysis

No correlations were found either between 2D and 3D echocardiography-derived parameters, or with any of the laboratory findings in HES patients. The logistic regression model identified presence of HES as an independent predictor of reduced MAFAC (hazard ratio (HR) 1.80, 95% CI of HR: 1.21 to 3.45, *p *< 0.05) and MAFS (HR 1.76, 95% CI of HR: 1.20 to 3.32, *p *< 0.05).

### 3.6 Reproducibility of 3D Echocardiography-Derived MA/TA Measurements

3D echocardiography-derived end-diastolic and end-systolic MA/TA dimensions were measured twice by the same observer (intraobserver agreement) and by two independent observers (interobserver agreement), the values were expressed as mean ± SD together with corresponding ICCs, the results are presented in Table 5.

**Table 5.** **Intra- and interobserver variability for mitral and tricuspid annular dimensions as assessed by three-dimensional echocardiography**.

	Intraobserver agreement		Interobserver agreement	
	Mean ± 2SD difference obtained by 2 measurements of the same examiner	ICC between measurements of the same examiner	Mean ± 2SD difference measured by 2 examiners	ICC between independent measurements of 2 examiners
Mitral annular dimensions				
End-diastolic MA diameter	0.02 ± 0.21 cm	0.94 (*p *< 0.0001)	0.03 ± 0.33 cm	0.95 (*p *< 0.0001)
End-diastolic MA area	–0.03 ± 0.98 ${\mathrm{cm}}^{2}$	0.95 (*p *< 0.0001)	0.02 ± 0.71 ${\mathrm{cm}}^{2}$	0.97 (*p *< 0.0001)
End-diastolic MA perimeter	–0.05 ± 0.75 cm	0.96 (*p *< 0.0001)	–0.08 ± 0.79 cm	0.96 (*p *< 0.0001)
End-systolic MA diameter	–0.02 ± 0.21 cm	0.96 (*p *< 0.0001)	0.03 ± 0.22 cm	0.96 (*p *< 0.0001)
End-systolic MA area	–0.03 ± 0.19 ${\mathrm{cm}}^{2}$	0.97 (*p *< 0.0001)	–0.05 ± 0.68 ${\mathrm{cm}}^{2}$	0.97 (*p *< 0.0001)
End-systolic MA perimeter	0.05 ± 0.89 cm	0.96 (*p *< 0.0001)	0.04 ± 0.63 cm	0.96 (*p *< 0.0001)
Tricuspid annular dimensions				
End-diastolic TA diameter	0.03 ± 0.31 cm	0.95 (*p *< 0.0001)	0.03 ± 0.43 cm	0.97 (*p *< 0.0001)
End-diastolic TA area	–0.03 ± 1.56 ${\mathrm{cm}}^{2}$	0.96 (*p *< 0.0001)	0.03 ± 0.88 ${\mathrm{cm}}^{2}$	0.97 (*p *< 0.0001)
End-diastolic TA perimeter	–0.06 ± 0.88 cm	0.95 (*p *< 0.0001)	–0.11 ± 0.76 cm	0.97 (*p *< 0.0001)
End-systolic TA diameter	–0.03 ± 0.54 cm	0.96 (*p *< 0.0001)	0.03 ± 0.58 cm	0.98 (*p *< 0.0001)
End-systolic TA area	–0.03 ± 0.55 ${\mathrm{cm}}^{2}$	0.97 (*p *< 0.0001)	–0.05 ± 0.55 ${\mathrm{cm}}^{2}$	0.95 (*p *< 0.0001)
End-systolic TA perimeter	0.06 ± 0.94 cm	0.96 (*p *< 0.0001)	0.05 ± 0.89 cm	0.96 (*p *< 0.0001)

Abbreviations: ICC, interclass correlation coefficient; MA, mitral annular; TA, tricuspid annular; SD, standard deviation.

### 3.7 Feasibility of 3D Echocardiography-Derived MA/TA Measurements

Two out of 17 HES patients (12%) were excluded from the study as image quality was poor (inadequate for visual qualitative analysis with or without artifacts). The overall feasibility of MA/TA measurements proved to be 88%.

## 4. Discussion

To our knowledge, the present study is the first in which MA and TA abnormalities in HES patients are presented by 3D echocardiography. Although both MA and TA showed signs of dilation in HES, MA abnormalities proved to be more pronounced, which accompanied by its functional impairment. Similar alterations for TA could not be detected. These results could highlight our attention on differences between left and right heart abnormalities in HES. Moreover, the novelty of the research was to demonstrate 3D echocardiography in the assessment of atrioventricular annuli on an easy-to-learn non-invasive way.

Aortic stiffness is increased in HES, but little is known about HES-related remodeling of heart chambers before the development of Loeffler endocarditis [7, 8, 9, 10, 19]. The novel echocardiographic technique, 3D echocardiography is a non-invasive method, and it seems to be optimal to quantify changes of atria and ventricles respecting the cardiac cycle [12, 13, 14]. A 3D speckle-tracking echocardiography-derived virtual 3D LV cast was used to detect the potential impairment in LV rotational mechanics demonstrating deteriorated apical rotation and twist and lack of LV twist (LV rigid body rotation) in 17% of HES cases mostly in the early necrotic phase [9]. LV longitudinal strain, one of the quantitative features of LV contractility was reduced as well suggesting subclinical functional impairment of LV function in HES [10]. Association was found between deteriorated LV function and elevated LA volumes respecting the cardiac cycle and increased total and active LA stroke volumes without impairment of LA emptying fractions. Moreover, LA circumferential strain was reduced as well [7]. The present findings provided more information about HES-related left heart abnormalities demonstrating dilated MA accompanied by its reduced function. The exact pathophysiology of these findings is not known, but subclinical involvement and infiltration of the walls of left heart chambers may be responsible. Moreover, the effects of different risk factors and aging could also play a role with haemodynamic effects of the aorta and its stiffened walls [19]. Moreover, the above mentioned functional abnormalities of the LV and LA could also have effects on each other resulting in MA dilation and functional impairment, as well [7, 9, 10].

Due to left heart abnormalities, changes may be present in the right heart as well despite absence of any cardiovascular symptoms. In a recent study, elevated RA volumes and mild RA functional abnormalities not affecting RA strains could be demonstrated in HES [8]. Although LA abnormalities found in HES were more significant compared with the RA, TA was found to be somewhat dilated, but obvious functional impairment could not be detected. Other studies should confirm our results and should evaluate HES-related RV abnormalities.

### Limitations

The following important limitations were present:

– Assessing any strains, rotational or dyssynchrony parameters of any chambers by 3D (speckle-tracking) echocardiography was not the aim of this study [12, 13, 14].

– HES is a rare disease, therefore we were able to collect clinical and 3D echocardiography-derived data of only relatively few HES patients [1, 2, 3, 4, 5, 6].

– There was a higher ratio of hypertension and hyperlipidemia in HES patients, which could affect result. However, comparative analysis between HES patients with vs. without hypertension did not find any differences in MA and TA data.

– In addition to the currently available technical development, lower temporal and spatial resolution features 3D echocardiography as compared to 2D echocardiography. This methodologic limitation could affect measurements [12, 13, 14].

## 5. Conclusions

The extent of the dilation of the MA is more pronounced than that of the TA in HES. MA functional impairment is present in HES.

## Data Availability

All data are available.
